# Exercise prescriptions for ischemic cardiomyopathy: a scoping review

**DOI:** 10.1007/s10741-025-10521-2

**Published:** 2025-05-07

**Authors:** Lida Koskina, Nicholas H. Huerta, Shiavax J. Rao, Ahmad Amin

**Affiliations:** 1 Medstar Health Georgetown University (Baltimore) Internal Medicine Residency Program, 201 E University Pkwy, Baltimore, MD USA; 2 Division of Cardiovascular Medicine, University of Virginia, Charlottesville, MD USA; 3 Division of Cardiovascular Medicine, Mayo Clinic, Rochester, MN USA

**Keywords:** Ischemic cardiomyopathy, Heart failure, Cardiac rehabilitation

## Abstract

This review explores the critical role of exercise as a non-pharmacological intervention in managing ischemic cardiomyopathy (ICM), a leading cause of heart failure. It highlights the profound cardiovascular benefits of exercise, such as improved cardiopulmonary parameters, decreased morbidity and mortality, and enhanced functional capacity. It also critically evaluates existing literature on the efficacy of various exercise types and intensities, including aerobic, resistance, and high-intensity interval training. There is a significant gap in current clinical guidelines, which lack specific exercise prescriptions tailored to the unique pathophysiology of ICM. By synthesizing data from both older and contemporary studies, this review highlights specific, evidence-based exercise regimens and promotes supervised cardiac rehabilitation programs. This review also addresses potential barriers to cardiac rehabilitation participation and proposes future directions, which include the use of technology to improve adherence and outcomes.

## Introduction

Cardiovascular disease (CVD) poses a significant global burden of morbidity and mortality and remains a leading cause of death across diverse populations. In 2021, CVD contributed to 20% of deaths in the United States (US) [1]. In the US, there is one MI every 40 s, which affects around 800,000 people annually[2]. Cardiovascular disease may lead to ischemic cardiomyopathy (ICM), which refers to a decrease in cardiac systolic function as a result of poor blood supply to the myocardium [3]. It is also noteworthy to mention that ICM may manifest independently of a preceding MI. This phenomenon is referred to as silent ischemia and highlights the complexity of ICM’s pathophysiology, where ischemic damage to the myocardium occurs without overt clinical symptoms of an MI. In addition to functional changes, ICM may also involve structural changes such as dilatation and fibrosis. Ischemic cardiomyopathy is the most common cause of heart failure [4]. While heart failure leads to significant morbidity and mortality in the population, it also imposes a notable strain on the healthcare system and is the second most common reason for hospitalizations in the US, responsible for 1,135,900 inpatient stays with an aggregate cost of 14.5 billion US dollars over a 1-year period [5].

Guideline directed medical therapy (GDMT) reduces heart failure hospitalization rates and provides substantial mortality and morbidity benefits in ICM. In addition to medical therapies, lifestyle interventions such as exercise can also have substantial benefits [6–10]. The 2023 AHA/ACC Guideline for the Management of Patients with Chronic Coronary Disease recommends ≥ 150 min/week of moderate-intensity aerobic activities or ≥ 75 min/week of high-intensity aerobic activities. This is a class I recommendation based on level A evidence [11]. They also recommend strength training ≥ 2 days/week, which is a class I recommendation based on level B-R quality of evidence [11]. In the supportive text, they go on to mention that high-intensity interval training is also an effective intervention [11]. The 2022 AHA/ACC heart failure guidelines similarly recommend exercise training as a class 1 recommendation based on A level of evidence; although, this recommendation is not specific to ischemic cardiomyopathy [12]. These guidelines do not go on to offer any more specific recommendations regarding exercise.

The evidence overwhelmingly supports exercise for the management of coronary artery disease (CAD) and heart failure with reduced ejection fraction (HFrEF). However, there are no specific recommendations for managing ICM, which is a vastly different clinical entity from stable CAD or nonischemic cardiomyopathy (NICM). Additionally, the guidelines do not define moderate-intensity exercise, high-intensity exercise, or high-intensity interval training. Furthermore, there is no recommendation on the frequency or individual duration of each exercise session. This presents a grand opportunity for improvement by providing individualized, evidence-based exercise regimens for each patient with ICM. If prescribed in a manner similar to medications (i.e., with a dose and frequency), individualized exercise recommendations may improve countless lives. A recent scientific statement from the American Heart Association and American Association of Cardiovascular and Pulmonary Rehabilitation offers more detailed exercise recommendations for CAD, which are likely to be included in the next set of guidelines [13]. The aim of this review is to evaluate the current evidence regarding exercise in the treatment of ICM so that clinicians may provide specific, evidence-based recommendations of exercise routines.

## Methods

**Fig. 1:**
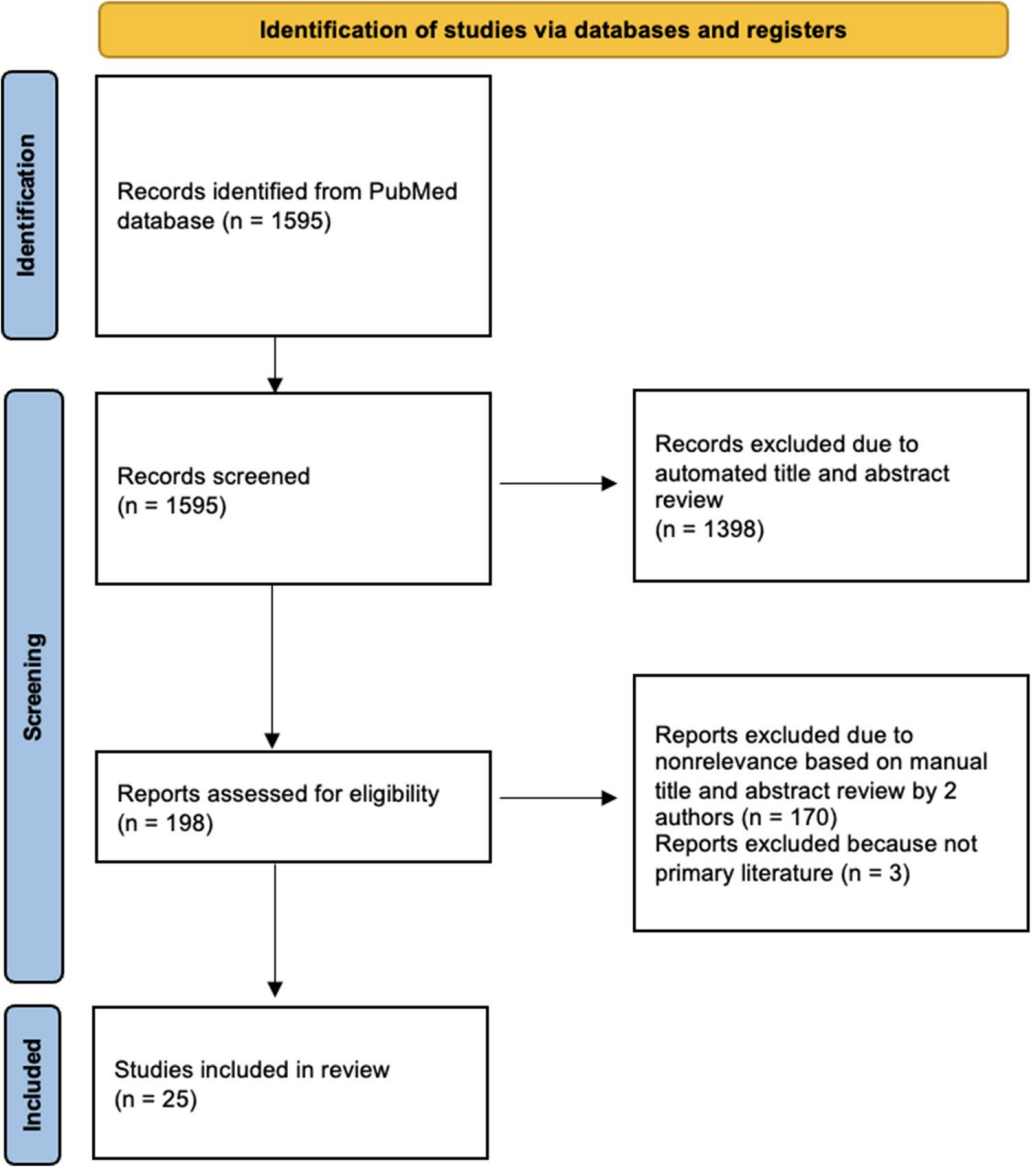
Flow diagram of the literature selection process

To identify and retrieve the relevant articles, a comprehensive search of the PubMed database was conducted. A combination of Medical Subject Heading (MeSH) terms and search words related to the topic of interest was used. MeSH and search terms included “ischemic cardiomyopathy,” “exercise,” “cardiac rehabilitation,” and “physical activity.” The initial search yielded 1595 articles. 1398 of the 1595 results were excluded due to an absence of the pre-specified search terms in the title and abstract. This left a final result of 198 studies. Only English language literature was included. There was no date range assigned to the search. Review articles and case reports were excluded. The titles and abstracts of the 198 articles were screened independently by two authors (L.K. and N.H.) to determine eligibility and relevance to the topic of interest. One hundred and seventy articles were excluded based on irrelevance to the topic of interest. Three articles were excluded on the basis of not being primary literature. A total of 25 studies were included in the review. Seventeen of the studies were exclusive to the ICM population and eight studies included mixed ICM and NICM populations. A flow diagram outlining the search strategy, screening, and data extraction is highlighted in Fig. 1.

## Results

### Neurohormonal and cardiopulmonary effects of exercise

The key results, including specific exercise protocols and outcomes, are summarized in Table 1. Several studies have shown the beneficial effects of exercise on cardiopulmonary parameters. On a fundamental level, exercise leads to changes in sympathovagal tone. Specifically, exercise leads to a decrease in norepinephrine and atrial natriuretic peptide levels, and improvements in heart rate variability [14, 15]. Further supporting alterations in sympathetic tone are decreases in both systolic and diastolic blood pressure, resting heart rate, and total peripheral resistance [15, 16]. Moreover, exercise training programs have been proven to cause a significant increase in both peak heart rate during exercise and heart rate reserve. However, this observation may be more profound in patients with nonischemic cardiomyopathy when compared to their ischemic counterparts [17]. Nevertheless, in ICM, exercise training still leads to an enhanced sympathetic response to exercise [18].

**Table 1:** Summary of key results of included studies

Author	Year	Study type	Intervention and outcome	Exercise intervention	Results
Kim C	2016	Retrospective	Aerobic exercise effect on graded exercise testing and LVEF change	30 min at 60–85% of HRR (using Karvonen formula) using a treadmill 3 times/week for 6 weeks. Also included 10-min warm up and 10-min cooldown Supervised sessions	pLVEF group with 7.4% increase in LVEF (*p* < 0.001) and 4 mL/kg/min increase in VO2peak (*p* = 0.002). rLVEF group with 7.9% increase in LVEF (*p* < 0.001) and 3.6 ml/kg/min increase in VO2peak (*p* < 0.001)
Halle M	2022	Randomized clinical trial	HIT, MCT, and RRE effects on LVEDD, LVEF, VO2peak	HIIT = four 4-min intervals at 90–95% of pHR with 3-min recovery periods at 60–70% of pHR done 3 times/week using treadmill or cycle ergometer MCT = 47 min of walking on treadmill at 70–75% of pHR 3 times/week for 12 weeks RRE = unsupervised exercise based on guidelines and preference Supervised sessions	No significant change in LVEDD, LVEF, or VO2peak after 12 weeks and no difference between exercise types. ICM with lower baseline VO2peak compared to NICM
Foccardi G	2021	Randomized clinical trial	Effects of text message after CR on GPAQ, submaximal iso-watt exercise testing, 30 s sit-to-stand test, arm curl test, and 7-point Likert scale	Daily text message at 0830 “The rehabilitation cardiology service reminds you to carry on with your physical activity program as indicated in the prescription.”	Increase in moderate physical activity time (Δ 244.7 min, *p* < 0.001). Reduction in sedentary behavior time (Δ − 77.5 min, *p* < 0.001). Significant decrease of exercise HR (*− *6.6 *p* < 0.01), SBP (− 9.6, *p* < 0.01, and RPE (− 1.2, *p* < 0.01). No significant difference in resting HR, SBP, or RPE. No difference in arm curl test. Improve 30 s sit-to-stand (Δ 2.2, *p* = 0.03)
Tyni-Lenné R	1997	Randomized crossover clinical trial in females*	8 weeks of knee extensor endurance training and 8 weeks of non-training. Measured effects on muscle metabolic capacity, exercise tolerance, and quality of life	Endurance leg training 3 times/week for 8 weeks. Exercises included bilateral dynamic knee extensions on an ergometer with 60 repeats/minute for 15 min. Intensity during first 4 weeks was 65% and during final 4 weeks was 75% of absolute baseline work rate (W) measured on the same ergometer. Training also included 6-min warm-up of walking and stretching leg muscles and 3-min cool-down Supervised sessions	Increased activity of citrate synthase (44%, *p* < 0.0001) and lactate dehydrogenase (23%, *p* < 0.002). Improved glycolytic oxidative capacity (23%, *p* < 0.002). Increased peak oxygen uptake (14%, *p* < 0.002) and peak work rate (43%, *p* < 0.001) during incremental exercise. Blood lactate during submaximal and recovery phase of exercise decreased (17%, *p* < 0.05). 6-min walk test improved (*p* < 0.03). Overall QOL improved (*p* < 0.01)
Legendre A	2021	Clinical Trial*	Effects of aerobic exercise training program on cardiorespiratory parameters (i.e. HR, workload, VO2, etc.) using CPET	60 min/day (30 min endurance and 30 min dynamic), 5 days/weeks for 4–6 weeks (total 20 sessions). 3 times/weeks bicycle endurance consisting of alternating 1-min bouts at 90% VO2peak with 4 min below VT. 2 times/week continuous cycling at VT for 30 min. Dynamic training included calisthenics and/or resistance training Supervised sessions	At rest, decrease in resting HR (*p* ≤ 0.001) and increase in VO2 (*p* < 0.01) At VT increase in power output (*p* < 0.0001), VO2 (*p* < 0.0001), VE (*p* < 0.0001), and O2P (*p* < 0.0001) At peak exercise increases in HR, workload (*p* < 0.0001), VO2 (*p* < 0.0001), VE (*p* < 0.0001), O2P (*p* < 0.0001), CP (*p* = 0.005), SV (*p* = 0.001), CI (p = 0.0001), QO2 (*p* = 0.0006), and DO2 (*p* = 0.006) and decreases in PvO2 (*p* < 0.0001) and SVR (*p* = 0.015) Resting LVEF rose from 28 to 32% (*p* = 0.001) and no significant differences between mitral E/e’ and sPAP
Whellan DJ	2001	Retrospective*	Associations between CR and survival	CR including aerobic training 3 times/week for 20–36 total sessions. Sessions done on a treadmill for ^3^30 min but usually ≤ 45 min at 60–80% of difference between resting HR and max HR	CR associated with lower adjusted 5-year mortality with early separation of Kaplan–Meier curve (91.9% vs. 63.8%, *p* < 0.002)
Chen SM	2019	Retrospective	Association between HFDMP and mortality and readmission	CR program from a physical therapist. Specifics not mentioned	ICM subgroup participating in HFDMP had lower readmissions (HR 0.13, *p* = 0.026). No difference in mortality in 1 year
Sponder M	2019	Prospective clinical trial*	Investigate effects of physical activity on biomarkers of myocyte ischemia and inflammation	Advised on at least 150 min/week of moderate intensity (65–75% max HR) or 75 min/week of vigorous intensity (76^ to 93% max HR). Strength training also allowed Exercise performed independently	Significant decrease in H-FAPB (*p* < 0.01) and increase in sST2 (*p* < 0.01). No change in suPAR levels
Caminiti G	2024	Randomized clinical trial	Compare effects of 12-week CT program on LA function. Exercise groups included CTLF, CTHF, and contemporary guidelines	Each session was 60 min with 10 min warm-up and 10 min cool-down CTHF was 3 sessions/week CTLF was 2 sessions per week Guideline group = home-based CTHF and CTLF = 40-min aerobic training on treadmill or bike using RPE to guide intensity with goal 13–14. Also included 20 min resistance training of 2 sets of 8 repetitions Movements included including leg press, leg extension, shoulder press, chest press, low row, and vertical traction CTLF and CTHF had supervised sessions done 8 AM to 11 AM	PALS significantly increased in both CTHF and CTLF group but was higher in CTHF group. PACS increased in CTHF group. SBP and resting HR decreased in CTHF and CTLF groups and was unchanged in guideline group
Vainshtein A	2011	Randomized in vivo rat study*	Analyze effects of 10-week exercise regimen on apoptotic susceptibility and response to acute oxidative stress in cardiac muscle	Voluntary running wheel with progressive loading. Unloaded for first 2 weeks then loaded with 50 g on weekly basis until 200 g reached on week 5. Then, this load maintained for 5 weeks	Training led to 52% increase in COX activity and reductions in AIF (32%, *p* < 0.05) and Bax/Bcl-2 levels (50%, *p* < 0.05). Attenuated JNK response to oxidative stress
Belardinelli R	1998	Randomized clinical trial	Investigate effects of 8-week exercise program on thallium uptake and contractile response to low-dose dobutamine	~ 60-min sessions 3 times per week for 8 weeks. 15-min warm-up and 40 min on cycle ergometer at 60% VO2peak Supervised exercise sessions	Exercise group with significant increases in VO2peak (4.2 mL/kg/min, *p* = 0.001), VT (3.1 ml/kg/min, *p* = 0.001), ventilation (16 L/min, *p* = 0.001), work rate (27 W, p = 0.001), and peak HR (7.5 BPM, *p* = 0.008) with decreases in resting HR (8 BPM, *p* = 0.001) Increased LVEF and decreased LVEDV at peak dobutamine Improvement in thallium activity in exercised cohort compared to control (*p* < 0.001)
Keteyian SJ	1999	Randomized clinical trial*	Analyze effects of 24-week exercise program on VO2peak, NE level, and QOL	3 times/week for 33 min using HR reserve method at 50% for first 2 weeks and increased as tolerated to 80%. Exercises included treadmills, stationary cycles, and arm ergometers	Larger increase in peak HR (9 BPM, *p* < 0.05) which was higher in those with chronotropic incompetence (12 BPM). VO2peak increased more in exercise group (204 mL/min, *p* < 0.05). Exercise led to greater reduction NE at rest and with exercise only in NICM and not in ICM. QOL unchanged
Belardinelli R	2006	Randomized clinical trial	Determine effects of 8-week exercise regimen on functional capacity, QOL, and readmissions in patients with ICD and CRT devices	3 times/week for 8 weeks. Each session was 60 min with 15-min warm-up, 40-min work, and 5-min cool-down. Work consisted of cycling on ergometer at 60% VO2peak Supervised sessions	Only trained patients had improvements in VO2peak (*p* < 0.01) and QOL (*p* < 0.001). CRT compared to ICD alone had greater improvements in VO2peak. Trained group had no ICD-related shocks (compared to 8 in control group) and had lower readmissions rate (67% vs. 45.4%, *p* < 0.0001)
Belardinelli R	1996	Randomized clinical trial	Determine if exercise can augment LV diastolic filling	3 times/week for 8 weeks. Each session was 60 min with 15-min warm-up, 40-min work, and 5-min cooldown. Work consisted of cycling on ergometer at 60% VO2peak Supervised sessions	Increased VO2peak (15%, *P* < 0.0001), work rate (15%, *p* < 0.005), peak early filling rate (10%, *p* < 0.02), and peak filling rate (11%, *p* < 0.03). Increased peak filling rate correlated with increased CI at peak exercise (*r* = 0.72, *p* < 0.0001)
Belardinelli R	1998	Randomized clinical trial	Analyze effects of exercise training on LV contractility with LDSE	3 times/week for 10 weeks. 10–15 min warm-up followed by 40 min on cycling ergometer at 60% VO2peak Supervised sessions	Exercise group with significant increases in VO2peak (28%), VT (31%), ventilation at peak exercise (33%), and work rate (21%). Resting LVEF similar between groups but LVEF at peak dobutamine levels increased in trained group (27%, *p* < 0.001)
Belardinelli R	1997	In vivo comparative human study	Determine kinetics of muscle oxygenation recovery (Vastus lateralis) in ICM and normal subjects after acute exercise	Incremental cycle ergometer at 6-min constant work rate protocol of 60% VO2peak	Muscle recovery and total body oxygenation from submaximal exercise is more delayed with cardiac dysfunction. Longer recovery VO2 and muscle oxygenation in ICM. Both VO2 and muscle oxygenation mean response times were inversely related to VO2peak (*r* = − 0.73 and − 0.52 respectively, *p* < 0.05)
Belardinelli R	1995	In vivo comparative human study	Use NIRS to determine if changes in skeletal muscle oxygen saturation during incremental exercise can reflect onset of anaerobic metabolism. Compared ICM to controls	Incremental work rate test until fatigue on cycle ergometer	Patients with ICM had earlier acceleration in muscle deoxygenation compared to healthy controls. ICM group also had a flatter increase in HR and SBP
Arahata K	2000	Prospective clinical trial*	Evaluate effects of exercise program in elderly patients with CHF NYHA III/IV	Unavailable	After exercise regimen, LVEF improved from 47.4 to 56% (*p* < 0.01) BNP decreased from 405 to 314 pg/ml (*p* < 0.01) quadriceps power increased from 0.77 to 0.97 Nm/kg (*p* < 0.05) maximum walking distance improved from 149 to 456 m, (*p* < 0.05)
McKelvie RS	1995	Prospective clinical trial	Compare hemodynamic effects during resistance exercise and endurance exercise using echocardiography	Resistance exercise consisted of 2 sets of 10 repetitions at 70% of 1RM Endurance exercise consisted of 5 min on the cycle ergometer at 70% of peak power output	Both exercises led to comparable increases in SBP. Leg press had higher DBP compared to cycling (98 mmHg vs. 86 mmHg, *p* < 0.05). Cycling had higher heart rate than leg press (107 vs. 86, *p* < 0.05). No differences observed in LVEDV or LVESV. Higher CO and stroke volume in cycling compared to leg press (9.3 L/min vs. 6.9 L/min, *p* < 0.05 and 87 mL vs 80 mL, *p* < 0.05, respectively). Similar LVEF between exercises
Shemesh J	1995	Retrospective	Assessment on long-term effects of rehabilitation training program on exercise performance, hemodynamics, and neurohormonal indexes. Compared those who participated to a matched group who did not participate	Program included either arm or calisthenics training. Arm training consisted of arm ergometry 2 times/week for 30 min of work at 80% of submaximal symptom-limited exercise HR. Calisthenics training was performed 2 times/week for 40 min with energy requirements of 2.2–7.8 kcal/min	Patients who participated in rehabilitation had lower resting HR (61 vs. 75 BPM, *p* < 0.05), lower resting NE levels (255 vs. 428 pg/mL, *p* < 0.05), and lower ANP levels (202 vs. 432 pg/mL, *p* < 0.05). Rehabilitated group showed greater HR increase (113% vs. 58%, *p* < 0.01) and better work capacity (6.9 vs. 3.7 METs, *p* < 0.05)
Chen Z	2018	Randomized in vivo rat study	Investigate the effects of aerobic exercise on Treg and Th17 cells in ICM animal model	Treadmill training 5 times/week for 12 weeks at 0º slope and 12 m/min	ICM rate model leads to significant increase in Th17 and decrease in Treg cells compared to control. Exercise led to decrease in Th17 and increase in Treg with overall decrease in Th17/Treg ratio (1.64 vs. 0.65, *p* < 0.01) which was comparable to the control group
Greenberg BH	2015	Randomized double-blind, placebo-controlled study	Assess safety and tolerability of omecamtiv mecarbil during symptom-limited exercise in patients with ICM	ETT consisted of treadmill testing using the Modified Naughton Protocol	No indications of increased likelihood of myocardial ischemia. 1 patient in placebo group stopped ETT due to angina and 0 in the treatment group stopped
Iliodromitis K	2023	Retrospective*	Assessment of physical activity in patient with WCD in patients with CHF	WCD measured daily steps	WCD was carried for average of 77 days. The total cohort saw improvements in LVEF over the study period (25.8% to 37.5%). This LVEF increase did not correlate with improvement in physical activity
Belardinelli R	2008	Randomized clinical trial	Assess if combination of trimetazidine and exercise training improve functional capacity and endothelial function more than exercise alone in ICM	Exercise training was performed 3 times/week for 8 weeks at 60% VO2peak	Trimetazidine and exercise compared to exercise alone led to significant improvements in VO2peak (25% vs. 15%, *p* ≤ 0.05), LVEF (18.4% vs. 12.9%, *p* ≤ 0.05), and endothelium-dependent dilation (8.4% vs. ~ 7%, *p* < 0.05)
Malfatto G	2003	Retrospective	Examine if etiology of CHF (NICM and ICM) influences autonomic response to CR	CR consisted of 3-month period of low-intensity exercise at 50% peak HR. Exercises included breathing exercises, free-body gymnastics, cycling, and treadmill	Before CR intervention the ICM group had higher sympathetic activity at rest and poor sympathetic response compared to NICM. CR led to enhanced sympathetic response in ICM group

*Not exclusive to ischemic cardiomyopathy population

*1RM* 1 repetition max, *AIF* apoptosis-inducing factor, *ANP* atrial natriuretic peptide, *BNP* brain natriuretic peptide, *CI* cardiac index, *CO* cardiac output, *COX* cytochrome c oxidase, *CP* cardiac power, *CPET* cardiopulmonary exercise testing, *CR* cardiac rehabilitation, *CRT* cardiac resynchronization therapy, *CTLF* combined training low-frequency, *CTHF* combined training high-frequency, *DBP* diastolic blood pressure, *DO2* oxygen diffusion, *ETT* exercise tolerance test, *GPAQ* global physical activity questionnaire, *H-FABP* heart-type fatty acid-binding protein, *HFDMP* heart failure disease management program, *HIIT* high-intensity interval training, *HR* heart rate, *HRR* heart rate reserve, *ICD* implantable cardioverter defibrillator, *ICM* ischemic cardiomyopathy, *JNK* c-Jun NH2-terminal kinase, *LA* left atrial, *LDSE* low-dose dobutamine stress echocardiography, *LVEDD* left ventricular end-diastolic dimensions, *LVEDV* left ventricular end-diastolic volume, *LVESV* left ventricular end-systolic volume, *MCT* moderate continuous training, *NE* norepinephrine, *NICM* non-ischemic cardiomyopathy, *NIRS* near-infrared spectroscopy, *O2P* oxygen pulse, *PALS* peak atrial longitudinal strain, *PACS* peak atrial contraction strain, *pHR* peak heart rate, *pLVEF* preserved left ventricular ejection fraction, *PvO2* partial venous oxygen pressure, *QO2* oxygen delivery, *QO2* max oxygen convection, *rLVEF* reduced left ventricular ejection fraction, *RPE* rate of perceived exertion, *RRE* recommendation of regular exercise, *SBP* systolic blood pressure, *sPAP* systolic pulmonary artery pressure, sST2 soluble isoform of suppression of tumorigenicity 2, *suPAR* soluble urokinase-type plasminogen activator receptor, *SV* stroke volume, *SVR* systemic vascular resistance, *VE* minute ventilation, *VO2peak* peak oxygen uptake, *VT* ventilatory threshold, *W* watts, *WCD* wearable cardioverter defibrillator

This enhanced sympathetic response leads to an improvement in cardiopulmonary function, as measured objectively by peak oxygen uptake (VO_2peak_). Belardinelli has published several studies showing improvements in VO_2peak_ [19, 20] which have also been corroborated by several other studies [14, 21, 22]. A contemporary study conducted by Legendre et al. investigated the effects of exercise on oxygen utilization via cardiopulmonary exercise testing (CPET) in patients with heart failure with reduced ejection fraction (HFrEF) secondary to ICM and dilated cardiomyopathy [23]. The authors identified three distinct responder groups based on oxygen utilization parameters. These groups included oxygen convection (QO_2peak_) plus diffusion (DO_2_), QO_2peak_ only, and DO_2_ only. QO_2peak_ was defined as the measure of the maximum amount of oxygen brought to the capillaries and was used as a central determinant of exercise capacity. DO_2_ measured the maximum diffusion capacity of oxygen in the muscle during peak exercise and was used as a peripheral determinant of exercise capacity. In this study, ICM was associated with a greater response in QO2 peak. Therefore, exercise seems to correlate with improved cardiopulmonary parameters, as measured by CPET.

### Effects of exercise on cardiac remodeling

Apart from improvement in cardiopulmonary parameters, exercise has also been linked with favorable cardiac remodeling. In a mixed population of NICM and ICM, Legendre et al. showed that resting left ventricular ejection fraction (LVEF) rose from 28 to 32% (*p* = 0.001), but there was no significant difference in systolic pulmonary artery pressure. Whether these differences have a prognostic value will need to be studied further [23]. Several other authors have supported the finding of improved EF (both at rest and in response to exertion) associated with exercise [24–26]. Moreover, Caminiti et al. demonstrated improvement in left atrial function and LVEF in patients with ICM who underwent a high-intensity training program when compared to the low-intensity and control groups[27]. These findings have been corroborated by multiple other studies that have previously described the beneficial effects of exercise in left ventricular reverse remodeling via imaging and molecular techniques [28–33].

### Predictors of response to exercise training

Imaging can also be helpful in identifying patients who would benefit from cardiac rehabilitation, despite unfavorable phenotypes such as prolonged ischemia time or persistent wall motion abnormalities at rest. For example, Belardinelli et al. showed that stress testing was sensitive in identifying patients with a hibernating myocardium and aiding in the selection of individuals who would benefit the most from an exercise training program [34]. Therefore, it is important to thoroughly screen patients and consider cardiac rehabilitation referral to a broader patient population than was originally thought.

### Effects of exercise on mortality and hospital readmission rates

The favorable cardiopulmonary effects of exercise seem to translate into improvements in hospitalization rates. Retrospective evidence in an ICM predominant population who had undergone cardiac rehabilitation demonstrates improved 5-year survival rates with early separation of the Kaplan–Meier curves when compared to patients who did not participate in cardiac rehabilitation [35]. This benefit persisted even after adjusting for multiple other variables that could account for increased survival. Additionally, they identified a dose-dependent relationship between survival and exercise, with patients participating in > 6 sessions showing the greatest benefit [35]. This dose-dependent effect was later corroborated by Beauchamp et al. in another long-term observational cohort study of patients with left ventricular dysfunction who attended cardiac rehabilitation [36]. Chen et al. also conducted a retrospective analysis demonstrating lower readmission rates in those who participated in a heart failure disease management program which included an exercise intervention [19]. Many meta-analyses of randomized controlled trials in mixed NICM and ICM populations also support a decrease in hospitalization [37–39].

Evidence also points towards the possibility of a mortality benefit of exercise in ICM. Belardinelli et al. investigated the effects of exercise on functional capacity and overall prognosis of patients. They were able to demonstrate an overall decrease in incident cardiac events and improvement in long-term outcomes in the trained group as compared to the control group [34]*.* The HF-ACTION trial was a randomized controlled trial in an evenly mixed ICM and NICM population. This study showed a reduction in all-cause and cardiovascular mortality, as well as all-cause and cardiovascular hospitalizations after prespecified adjustments [40]. However, there is some disagreement between meta-analyses in terms of potential mortality effects. For example, a meta-analysis with relatively short-term follow-up (6 months to 1 year) failed to find a mortality benefit. However, there was a nonsignificant trend towards a mortality benefit at longer follow-up [37]. This trend aligns with other meta-analyses which show a significant mortality benefit at longer follow-up [38, 39]. It should be noted that these meta-analyses are in general HFrEF populations irrespective of etiology. Further evidence is needed in ICM-specific populations to parse out the effects of exercise on mortality.

### Effects of exercise on overall quality of life

The results on overall quality of life after exercise interventions have been contradictory. For example, Keteyian et al. did not find a correlation between improved exercise capacity and quality of life, but this was in a mixed population of NICM and ICM [17]. Similar were the results of Wilson et al., who did not find a significant difference in quality of life, as measured by questionnaires. Although, participants did report an overall improvement in their well-being as a result of the exercise program [21].

Contradictory were the results reported by Kavanaugh et al., where participants of an exercise training program reported significant improvement in their quality of life [22]. Additionally, Belardinelli et al. showed that both functional capacity and quality of life improved with exercise in their cohort [19]. Other studies have also identified improvements in the perception of general well-being, the perception of symptoms, and anxiety in patients who completed a structured cardiac rehabilitation program [25, 41]. Additionally, meta-analyses including all etiologies of HFrEF demonstrated significant improvements in quality of life [37–39].

### Exercise protocols and their effects on outcomes

Overall, different types of exercise appear to have similar beneficial effects in ICM. Table 1 provides a detailed description of the exercise interventions and various outcomes for each study identified using the search criteria. This table should be used as a guide to prescribing exercise in ICM. Most studies did not directly compare exercise protocols. However, studies identified with the search strategy contain a mixture of protocols which include low-intensity, moderate-intensity, high-intensity, and resistance training. McKelvie et al. showed that both aerobic exercise and anaerobic exercise had comparable results in several hemodynamic parameters [16]. The Study of Myocardial Recovery after Exercise Training in Heart Failure (SMARTEX-HF) trial investigated the effect of moderate-continuous exercise, high-intensity interval training (HIIT), and recommendation of regular exercise in patients with HFrEF. They categorized patients based on heart failure etiology and compared ischemic vs. non-ischemic cardiomyopathy in a predefined subgroup analysis. They found no difference in left ventricular remodeling, EF, or peak oxygen consumption between patients with ischemic vs non-ischemic cardiomyopathy, but ICM patients had lower baseline and follow-up VO_2_ after 12 weeks. The authors noted that the ICM group was significantly older and more likely to be on statin therapy, which may be an explanation for the observation. However, both moderate-continuous exercise and HIIT were superior to the recommendation of regular exercise. High-intensity interval training led to a slight improvement in left ventricular end-diastolic volume at the end of the 12 weeks supervised program, but this improvement did not persist at the 52-week follow-up [42]. These short-term improvements align with other literature showing a brief period of mild-to-moderate exercise was enough to induce improvement in exercise capacity and quality of life in patients with ICM and left ventricular dysfunction [25].

### Debates and controversies

**Fig. 2:**
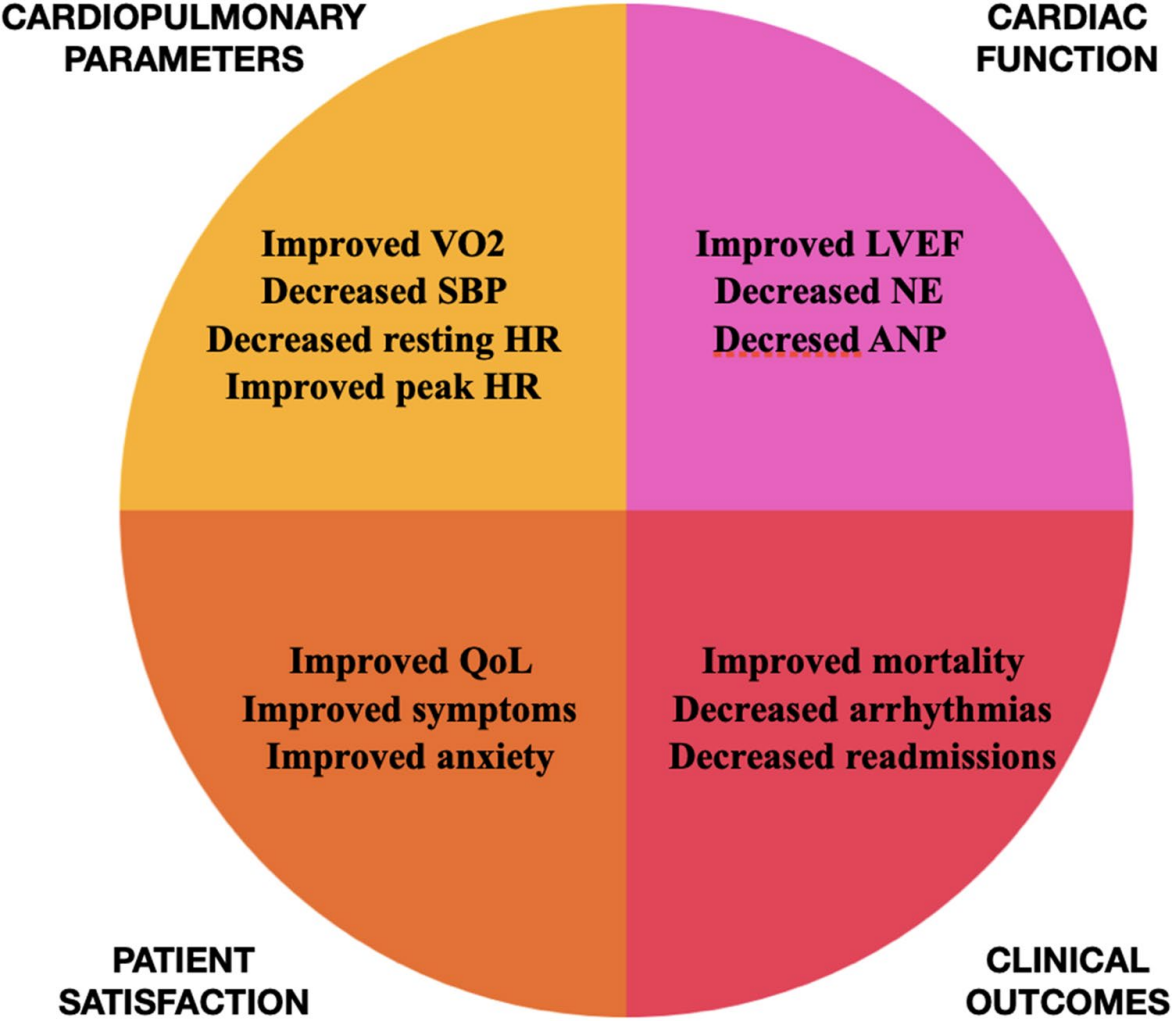
Overview of the benefits of exercise in ischemic cardiomyopathy

Despite the apparent beneficial effects of exercise, there has been controversy regarding its safety in this patient population. For instance, Whellan et al. voiced concerns regarding the safety of exercise in patients with ICM and left ventricular dysfunction based on results from previous studies demonstrating increased adverse events and malignant arrhythmias [35]. However, a study by Belardinelli et al. showed that there were no recorded malignant arrhythmias, ICD shocks, or deaths in patients who completed an exercise program. On the contrary, patients in the non-exercise group developed ventricular arrhythmias leading to ICD shocks and hospitalizations. The authors proposed that exercise-induced reduction in neurohormonal activation, as measured by lower epinephrine and norepinephrine levels, was the primary mechanism to explain this observation [19]. Kim et al. also showed that cardiac rehabilitation was both beneficial and safe for patients with ischemic cardiomyopathy and a reduced ejection fraction [24]. Figure 2 summarizes the major beneficial effects of exercise in ICM identified in the literature.

## Discussion

Patients with ICM seem to have a favorable response to exercise based on the studies with an ICM cohort. This was demonstrated via improvement in sympathovagal tone modulation, heart function biomarkers, cardiopulmonary function parameters, imaging findings, and quality of life [15, 16, 24, 25, 27]. Moreover, some studies included a mixed ICM and NICM cohort. In most of these studies, there was no direct comparison between these two patient populations, but the authors suggested an overall positive response to exercise in patients with cardiomyopathy [17, 43]. Halle et al. specifically compared the ICM and NICM cohorts in a prespecified subgroup analysis and found that there was no significant difference in the beneficial effects of exercise on cardiac remodeling, LVEF, or exercise capacity based on the etiology of the cardiomyopathy [42]. The aforementioned studies are presented in Table 1, which outlines the individual exercise protocols and results and highlights the studies with a mixed population of ICM and NICM with an asterisk.

Apart from its beneficial effects on patients with an established cardiomyopathy, exercise can also substantially decrease morbidity and mortality from the totality of cardiovascular causes. A cochrane review from 2016 found that exercise-based cardiac rehabilitation led to a 26% decrease in CVD mortality and an 18% decrease in hospital admissions [6]. Another review from 2019 found that exercise interventions in patients with heart failure led to a 43% reduction in heart failure hospitalizations, a 23% reduction in all-cause hospitalizations, and improvements in quality of life [7]. Additionally, in a population with angina pectoris and stable coronary artery disease, a 12-month exercise routine compared to percutaneous coronary intervention (PCI) resulted in superior event-free survival, higher exercise capacity, and improved cost-effectiveness [8]. A meta-analysis of randomized controlled trials found that exercise interventions led to a 27% reduction in all-cause mortality, a 31% reduction in total cardiac mortality, and reductions in total and LDL cholesterol in a population with coronary artery disease [9]. A large observational study found that patients with CAD at a low fitness level experienced a 29% increased risk of all-cause mortality compared to those at an elite fitness level, which was quantified by metabolic equivalents (METs) on exercise treadmill testing. Notably, in the entire cohort, low cardiorespiratory fitness compared to elite cardiorespiratory fitness was associated with a 400% increased risk of all-cause mortality (HR = 5.04) [10]. Thus, it seems the beneficial effects of exercise can extend beyond the ICM population and translate into an increase in overall survival in patients with CAD and CVD risk factors.

Despite the evidence on the benefits of cardiac rehabilitation, enrollment and adherence to these training programs has been traditionally low [44, 45]. Several barriers have been identified, some of which are modifiable. Among the non-modifiable barriers are low socioeconomic status, financial constraints, distance to facility, lack of transportation, lack of family or social support, and other patient-related factors such as psychological status, low self-esteem, and lack of motivation to improve one’s health [46]. These factors can be difficult to overcome. Other barriers that have been previously identified in various studies include older age, ethnic minorities, the presence of multiple comorbidities, female sex, and history of NSTEMI [45, 47]. Moreover, patients who had an intervention while in the hospital were more likely to be referred to cardiac rehabilitation [48]. However, even among patients who had an intervention, undergoing cardiac surgery was most strongly associated with an outpatient cardiac rehabilitation referral when compared to patients who had a percutaneous intervention [49]. A randomized clinical trial conducted by Gaalema et al. found that involving case managers in the care of these patients, as well as offering financial incentives for cardiac rehabilitation completion, led to a significantly higher adherence [50]. Even though offering financial incentives may not be a viable solution for many institutions, educating patients regarding the benefits of cardiac rehabilitation during hospitalization, as well as involving case managers and other healthcare providers that can support patients during their transition to the outpatient setting, is crucial to increasing enrollment in and adherence to a longitudinal training program.

Cardiac rehabilitation should be pursued under the direct supervision of trained professionals in a controlled environment to maximize benefits and avoid adverse events. Attending a supervised rehabilitation program ensures continuous patient monitoring, thus enhancing safety and potentially alleviating patients’ fears of exercise after a cardiovascular event [25, 41]. It is paramount that patients continue to exercise safely on their own after graduating from a structured exercise program. Of note, some of the beneficial effects of exercise were transient and only observed immediately after the supervised exercise period. This suggests that continued supervision and support lead to more benefits. Based on the literature, moderate aerobic exercise on a treadmill or stationary bicycle, 3–4 times per week, at 60–70% of peak heart rate for 20–30 min is a reasonable regimen that can be used for the majority of patients [25, 42]. However, it is important to consider alternatives, such as anaerobic training or other stationary exercise options for patients with mobility restrictions or those who have a higher frailty index. These could include 10–15 repetitions of stationary exercises using light weights or dumbbells, or the use of resistance bands, 3–4 times per week for 10–15 min [16]. Tailoring the exercise regimen to an individual patient will accomplish greater adherence and patient satisfaction while minimizing adverse events. Table 1 should be used as an evidence-based guide to prescribing exercise in ICM. A gradual increase of exercise intensity, as well as the incorporation of monitoring devices such as smartphones or watches, can further enhance safety during exercise and allow patients to feel more empowered during these activities [51].

Even though exercise has been proven generally safe in multiple studies, adverse events may still occur, and every precaution should be taken to minimize them. Adequate time for myocardial recovery after a coronary event should be allowed before initiating exercise [19]. It is also important to consider arrhythmia as a major complication early in the training process before neurohormonal dampening and reverse remodeling have occurred [35]. Any relevant comorbidities that can impact the ability of patients with ICM to exercise should be addressed in a multi-disciplinary manner to optimize them for cardiac rehabilitation. Finally, musculoskeletal injury should be avoided by encouraging patients to attend a supervised exercise program at the initial stages for the added benefit of education as well as a gradual increase in exercise intensity under close monitoring.

## Future directions

With the advancement of technology, there are many opportunities to improve adherence to cardiac rehabilitation for patients with ICM. For example, surveillance of physical exercise through the use of a wearable defibrillator can lead to better clinician monitoring, adjustment of medications, and exercise prescriptions in mixed NICM and ICM populations [52]. Moreover, interventions as simple as text messages can significantly increase adherence to cardiac rehabilitation and improve overall functional status and prognosis in patients at risk of cardiac events [53]. The use of electronic devices, such as smartphones or smartwatches, has shown great promise in increasing adherence to training programs and improving peak VO_2_ and 6-min walking distance [51]. Given recent advances in artificial intelligence (AI) and its applicability to health care, it is also possible that AI-guided programs may be capable of creating easily accessible exercise regimens (Table 2). Incorporating technology in cardiac rehabilitation modalities can therefore increase access to this intervention and lead to improved adherence and benefits.

**Table 2:** Output from GPT-4.0 based on the input “Create an exercise routine for someone with ischemic cardiomyopathy based on the current research”

Exercise component	Details
Pre-exercise considerations	**Medical clearance:** obtain from a cardiologist **Baseline assessment:** cardiac function evaluation (e.g., stress testing) **Monitoring:** use a heart rate monitor or wearable device
Warm-up	**Frequency:** 3–5 times per week **Duration:** 20–40 min **Intensity:** moderate (50–70% of maximum heart rate; RPE 11–13) **Types:** walking, stationary cycling, swimming/water aerobics
Aerobic exercise	**Frequency:** 3–5 times per week **Duration:** 20–40 min **Intensity:** moderate (50–70% of maximum heart rate; RPE 11–13) **Types:** walking, stationary cycling, swimming/water aerobics
Strength training	**Frequency:** 2–3 times per week (with rest days) **Duration:** 15–20 min **Intensity:** light to moderate weights, 10–15 reps **Types:** bodyweight exercises, resistance bands, light dumbbells
Flexibility and balance	**Frequency:** 3–4 times per week **Duration:** 10–15 minu **Types:** stretching (major muscle groups), balance exercises, yoga, Tai Chi
Cooldown	**Duration:** 5–10 min **Activity:** gradual decrease in intensity (slow walking, cycling) followed by deep breathing exercises
Important considerations	**Hydration:** ensure proper hydration **Medication:** consider timing with exercise **Symptoms monitoring:** stop if chest pain, shortness of breath, dizziness, or palpitations occur **Rest and recovery:** allow sufficient rest between workouts **Gradual progression:** increase duration and intensity gradually
Follow-up	**Regular check-ups:** adjust exercise plan as needed with healthcare provider

## Limitations

The limitations of this study are inherent to its literature review design. Selection bias or failure to identify the totality of available studies could have influenced our results and perception of the beneficial effects of exercise in ICM. Furthermore, the use of a single database for article identification could have reduced the pool of available evidence. However, this risk is mitigated by the fact that PubMed remains one of the most comprehensive databases for medical literature.

Moreover, several studies examined the effects of exercise on HF patients without differentiating or restricting analysis to ICM patients. However, most of the studies included in this review had a predominantly ICM population; thus, it is reasonable to extrapolate the final conclusion based on these results. Finally, only one study compared ICM and NICM patients directly; therefore, it is difficult to ascertain whether either population would benefit more from an exercise training program based on our results. Further studies are needed on this topic to further characterize the effects of exercise based on the etiology of cardiomyopathy.

## Conclusion

The evidence suggests that exercise leads to improvements in sympathovagal tone, biomarkers, cardiopulmonary function parameters, quality of life, hospitalization rates, and mortality in patients with ICM. Given the growing prevalence of this disease, it is important to conduct studies focusing on this population, as the underlying pathophysiology differs dramatically from NICM. Despite the benefits, enrollment and participation in cardiac rehabilitation have remained low. Several barriers may preclude patients from adhering to an exercise regimen. However, raising awareness of the beneficial effects of exercise, as well as incorporating new technologies in delivering these interventions, can lead to greater patient participation. It is important to treat exercise prescriptions like any other medication prescription in order to avoid confusion and adverse events in patients who cannot tolerate certain activities. Participation in structured cardiac rehabilitation programs, at least in the initial phase, is therefore paramount in increasing the efficacy and safety of exercise regimens.

## Data Availability

No datasets were generated or analysed during the current study.
